# Serum Gremlin 1 Remains Elevated Following Successful Direct-Acting Antiviral Therapy for Chronic Hepatitis C

**DOI:** 10.3390/v18070773

**Published:** 2026-07-14

**Authors:** Kilian Weigand, Georg Peschel, Martina Müller, Christa Buechler

**Affiliations:** 1 Department of Internal Medicine I, Gastroenterology, Hepatology, Endocrinology, Rheumatology, and Infectious Diseases, University Hospital Regensburg, 93053 Regensburg, Germany; kilian.weigand@gk.de (K.W.); georg.peschel@klinikum-ffb.de (G.P.); martina.mueller-schilling@klinik.uni-regensburg.de (M.M.); 2 Department of Gastroenterology, Gemeinschaftsklinikum Mittelrhein, 56073 Koblenz, Germany; 3 Department of Internal Medicine, Klinikum Fürstenfeldbruck, 82256 Fürstenfeldbruck, Germany

**Keywords:** direct-acting antivirals, hepatitis C, liver cirrhosis, genotype, gremlin 1

## Abstract

Background: Gremlin 1 is a fibrotic protein that increases in the liver during fibrosis. There is also evidence that gremlin 1 expression is upregulated in the livers of patients with hepatitis C virus (HCV) infection. Since gremlin 1 is a secreted protein, this study is the first to examine whether serum gremlin 1 could serve as a noninvasive marker of liver fibrosis in patients with chronic HCV infection. The study also examined whether viral clearance with direct-acting antivirals (DAAs) affects circulating gremlin 1 levels. Methods: Serum gremlin 1 levels were measured in 30 controls and 114 patients with chronic HCV, as well as in 60 HCV patients for whom serum was available prior to therapy and at four and 12 weeks after therapy initiation and 12 weeks post-therapy. Results: Compared with 30 healthy controls, patients with HCV exhibited significantly higher serum gremlin 1 levels. Among patients with cirrhosis, defined by noninvasive scores or ultrasound, serum gremlin 1 levels were only modestly elevated. Serum gremlin 1 levels positively correlated with markers of inflammation and aminotransferase levels in HCV patients. During antiviral therapy, serum gremlin 1 levels transiently increased four weeks after therapy initiation and returned to pretreatment levels by week 12. Even though patients were effectively cured of the virus, patients still exhibited higher serum gremlin 1 levels compared with controls at 12 weeks post-therapy. Conclusions: Our findings indicate that chronic HCV infection is associated with elevated serum gremlin 1 levels, which do not return to normal shortly after viral cure. In contrast, serum gremlin 1 concentrations do not provide a reliable indicator of fibrosis stage in patients with chronic HCV infection.

## 1. Introduction

Chronic hepatitis C virus (HCV) infection induces persistent hepatic inflammation, which promotes the development and progression of liver fibrosis [1,2]. The virus also disrupts host lipid metabolism to support its replication, thereby contributing to hepatic fat accumulation [3,4]. Hepatic steatosis is common in individuals with HCV, particularly among those infected with genotype 3 [3]. In parallel, the increasing prevalence of overweight and obesity further exacerbates steatosis [5]. Both HCV infection and excess body weight are linked to insulin resistance, which amplifies hepatic lipid deposition [3]. Metabolic factors such as obesity, insulin resistance, chronic inflammation, and dysregulated adipokine signaling play a central role in the progression of liver disease [6].

Eradication of HCV with direct-acting antivirals (DAAs) is associated with reduced fasting insulin levels, indicating improved glucose regulation [7]. However, liver steatosis mostly persists after viral cure [8]. Viral clearance also leads to rapid attenuation of hepatic inflammation and partial regression of fibrosis; however, full reversal of fibrotic changes may take several years [9,10,11,12].

Non-invasive approaches for assessing liver fibrosis are now widely used. Shear wave elastography (SWE) provides a quantitative measure of liver stiffness and is considered reliable for fibrosis staging in patients with HCV [13]. The fibrosis-4 (FIB-4) index—derived from age, alanine aminotransferase (ALT), aspartate aminotransferase (AST), and platelet count—is also a validated tool for identifying advanced fibrosis [14]. Notably, following successful antiviral therapy, decreases in ALT and AST levels can lead to lower FIB-4 scores and SWE values at sustained virological response 12 (SVR12), primarily reflecting reduced inflammatory activity rather than fibrosis resolution [11,15].

Gremlin 1, a known antagonist of bone morphogenetic proteins (BMP2, BMP4, and BMP7), modulates BMP signaling by binding to these ligands and suppressing their downstream activity [16]. BMP7, but not BMP2 and BMP4, has been shown to promote HCV entry into hepatocytes [17]. However, another study found that BMP7 suppresses HCV replication in hepatocytes [18]. HCV patients had higher serum BMP7 levels than healthy controls, but these levels did not further increase in patients with HCV-related cirrhosis [19]. BMP2 expression in the livers of patients who responded to pegylated interferon plus ribavirin therapy was lower than in those who did not respond [20]. Sinusoidal endothelial cell-released BMP4 increased HCV replication, and BMP4 protein was induced in the livers of HCV patients compared to controls [21]. The roles of BMPs 2, 4, and 7, and of gremlin 1, as a BMP antagonist, in HCV infection are currently unclear.

Elevated gremlin 1 levels have been associated with impaired insulin sensitivity and systemic inflammation, both of which can contribute to the metabolic disturbances observed in HCV infection [4,22,23,24]. Experimental evidence further indicates that gremlin 1 aggravates high-fat diet–induced hepatic steatosis in murine models [25]. In the liver, increased gremlin 1 expression has been linked to insulin resistance, and more recent findings suggest a role in promoting hepatocyte senescence, a process associated with fibrogenesis [22,26].

Activation of hepatic stellate cells (HSCs) is a key driver of liver fibrosis [27]. Gremlin 1 expression is upregulated in activated HSCs and in fibrotic liver tissue in animal models [28]. Additionally, gremlin 1 has been shown to enhance the expression of transforming growth factor beta, a central profibrotic cytokine that stimulates extracellular matrix production [27,29]. Silencing of gremlin 1 via siRNA inhibits HSC activation and attenuates fibrosis in rodent models [30]. However, not all findings are consistent; antibody-mediated neutralization of gremlin 1 activity has been shown not to significantly affect liver inflammation or fibrosis [23].

At the transcriptional level, gremlin 1 mRNA is markedly increased in activated HSCs, whereas its expression is minimal in healthy liver tissue but induced under fibrotic conditions [28,29]. Upregulation of gremlin 1 has been reported in fibrosis associated with metabolic dysfunction–associated steatohepatitis (MASH) in both humans and animal models, as well as in the livers of patients with chronic HCV infection [23,31]. Notably, hepatic gremlin 1 protein levels in HCV cohorts have not shown a clear association with fibrosis stage, age, or sex [31].

Beyond fibrosis, gremlin 1 has also been implicated in hepatocarcinogenesis. In hepatocellular carcinoma (HCC), cancer-associated fibroblasts secrete gremlin 1 via exosomes, promoting epithelial–mesenchymal transition in hepatocytes [32]. Elevated gremlin 1 expression in cirrhotic liver tissue has therefore been proposed to contribute to HCC development [33].

Circulating gremlin 1 levels have been examined in several clinical contexts. In females, serum levels appear to increase with age rather than reflect adiposity or metabolic dysfunction per se [34]. Elevated circulating gremlin 1 levels have also been observed in severe COVID-19 cases requiring intensive care [35]. In addition, higher serum gremlin 1 concentrations have been associated with key features of metabolic liver disease, including steatosis, inflammation, and fibrosis [22].

Although existing evidence indicates that hepatic gremlin 1 expression is elevated in fibrotic liver disease, it remains unclear whether circulating gremlin 1 can serve as a reliable non-invasive biomarker of fibrosis in chronic HCV infection. Therefore, the present study aimed to evaluate the association between serum gremlin 1 levels and markers of liver disease severity. In addition, the study examined the impact of DAA therapy on circulating gremlin 1 concentrations to determine the effect of viral eradication. It should be noted that serum gremlin 1 levels have not been measured in chronic HCV, nor has the effect of viral cure been studied so far.

## 2. Materials and Methods

### 2.1. Study Cohorts

This prospective study included patients with chronic HCV infection from 1 September 2014 to 27 February 2017 and was conducted in the Department of Internal Medicine I at the University Hospital Regensburg. Participants were patients indicated for treatment with DAAs.

This study initially included 217 patients with chronic HCV infection (Figure S1). At the time of the current analysis, serum samples from 114 patients who had not been treated with any drugs were available. Serial serum samples collected before treatment, at weeks 4 and 12 of therapy, and 3 months after the end of treatment were available for 60 patients. In addition, serum samples obtained at SVR12 were available for 85 of the patients (Figure S1).

The 60 patients with serial serum samples received the following treatment regimens: sofosbuvir/daclatasvir (n = 25), sofosbuvir/daclatasvir/ribavirin (n = 2), sofosbuvir/ledipasvir (n = 14), sofosbuvir/velpatasvir (n = 3), ledipasvir/sofosbuvir/ribavirin (n = 3), sofosbuvir/ribavirin (n = 6), or dasabuvir/ombitasvir/paritaprevir/ritonavir (n = 7) [36,37,38]. The patients were aged 18 years or older and had not previously received treatment for HCV. Individuals with decompensated liver cirrhosis, co-infection with the hepatitis B virus, or human immunodeficiency virus were excluded from the study.

The study included 30 healthy controls with a median age of 56 (21–67) years, similar to that of the patients (*p* = 0.450). There were 19 female and 11 male controls, and sex did not differ between the controls and patients with HCV (*p* = 0.102).

Laboratory measurements were supplied by the Institute of Clinical Chemistry and Laboratory Medicine and the Institute of Clinical Microbiology and Hygiene at the University Hospital Regensburg.

### 2.2. Ultrasound Examination and Calculation of the Fibrosis Score

The diagnosis of cirrhosis via ultrasound is based on the presence of a nodular liver surface, a reduced liver size, and coarse liver parenchyma [39]. The fibrosis-4 (FIB-4) score is a recognized non-invasive measure of fibrosis [40,41,42]. The established cut-off values for the FIB-4 score indicate that a score greater than 3.25 suggests advanced fibrosis; a score below 1.3 indicates a low probability of advanced fibrosis in patients under 65 years of age; and a score below 2 indicates no fibrosis in patients over 65 years of age [41]. This score has been initially defined in patients with non-alcoholic fatty liver disease [41], but can be used for chronic liver diseases of different etiologies [14,43]. Tissue elastography was quantified by dynamic shear-wave elastography. The devices used in this study included the GE LOGIQ E9 (1–6 MHz probe) (GE HealthCare, Düsseldorf, Germany), the Siemens Acuson S2000 (1–5 MHz probe) (Siemens Healthineers AG, Forchheim, Germany) and the Siemens Acuson S3000 (1–6 MHz probe). A median value was derived from 8 to 12 valid measurements, and fibrosis grading was conducted using cutoff values established by Colombo et al. [44].

### 2.3. Gremlin 1 Enzyme-Linked Immunosorbent Assay (ELISA)

Serum aliquots were stored at −80 °C and were thawed immediately prior to use. Serum was diluted 1:100 for gremlin 1 measurement with the human gremlin 1 ELISA Kit (Catalog #: BYT-ORB776964; Biozol, Hamburg, Germany). All serum samples were measured in duplicate, and the mean value was used for further calculation.

### 2.4. Statistical Analysis

Serum gremlin 1 levels in patients with chronic HCV were not normally distributed (Shapiro–Wilk test, *p* < 0.001). The results are presented as boxplots, which illustrate the minimum, maximum, median, first quartile, and third quartile. Small circles above or below the boxes indicate outliers. The data in the tables are presented as medians, with the minimum and maximum values in parentheses. The nonparametric Mann–Whitney U test and the Kruskal–Wallis test were employed to compare two independent groups and more than two independent groups, respectively (SPSS Statistics 31.0; IBM Corp., Armonk, NY, USA, 2019). To compare paired samples, the Wilcoxon test or the Friedman test was used (SPSS Statistics 31.0). Spearman’s rank correlation was used to analyze associations. Multiple linear regression to predict serum gremlin 1 levels was also performed in SPSS (SPSS Statistics 31.0). Data were not adjusted for multiple comparisons. A value of *p* < 0.05 was regarded as significant.

## 3. Results

### 3.1. The Relationship Between Serum Gremlin 1 Levels and Age, Body Mass Index, Gender, Liver Steatosis, and Diabetes in Patients with Chronic HCV Infection and Comparison with Healthy Controls

This study included 114 patients with chronic HCV infection, whose characteristics are summarized in Table 1.

**Figure 1:**
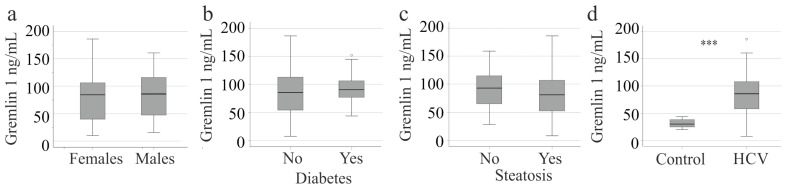
Serum gremlin 1 levels of patients with chronic hepatitis C virus (HCV) stratified for sex, diabetes, and liver steatosis, and comparison to healthy controls: (**a**) Serum gremlin 1 levels of female and male HCV patients; (**b**) Serum gremlin 1 levels of HCV patients with and without diabetes; (**c**) Serum gremlin 1 levels of HCV patients with and without steatosis; (**d**) Serum gremlin 1 levels of controls and HCV patients. The small circles above the boxes mark outliers. *** *p* < 0.001.

In this cohort, serum gremlin 1 levels of the 52 females and the 62 males were comparable (Figure 1a). Correlations of serum gremlin 1 with the body mass index (BMI) (r = −0.082, *p* = 0.427) and age (r = −0.044, *p* = 0.645) were not significant. The 19 HCV patients with diabetes had serum gremlin 1 levels similar to those of non-diabetic HCV patients (*p* = 0.071) (Figure 1b). Fifty-eight patients had liver steatosis according to B-mode ultrasound, but serum gremlin 1 did not differ from patients without liver steatosis (*p* = 0.947) (Figure 1c).

Serum gremlin 1 in the 30 controls was significantly lower compared to patients with HCV (Figure 1d). In the controls, serum gremlin 1 did not correlate with age (r = 0.001, *p* = 0.995) and was similar between sexes (*p* = 0.846).

### 3.2. The Relationship Between Serum Gremlin 1 Levels and Liver Fibrosis in Patients with Chronic HCV Infection

**Figure 2:**
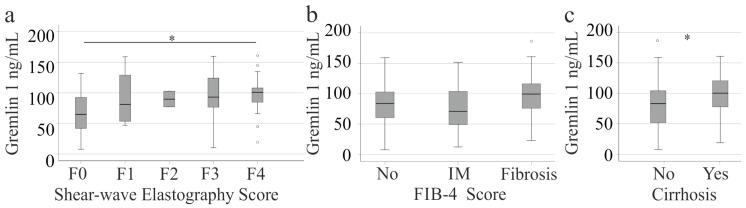
Serum gremlin 1 levels in patients with chronic hepatitis C, stratified by fibrosis: (**a**) Serum gremlin 1 levels of patients with increasing fibrosis stages defined by shear-wave elastography. (**b**) Serum gremlin 1 levels in patients with increasing fibrosis stages defined by the FIB-4 score, where No indicates a low probability of fibrosis and fibrosis refers to advanced fibrosis. (**c**) Serum gremlin 1 levels in patients with and without liver cirrhosis defined by ultrasound. The small circles above and below the boxes mark outliers. * *p* < 0.05.

We used noninvasive measures to evaluate liver fibrosis: shear-wave elastography (SWE) and the fibrosis-4 (FIB-4) score. SWE indicated 43 patients with F0, 20 with F1, 2 with F2, 15 with F3, and 34 with F4 fibrosis. Serum gremlin 1 was higher in patients with F4 fibrosis than in those with F0 fibrosis, but did not otherwise differ between the subgroups (Figure 2a). According to the FIB-4 score, 40 patients had a low probability for fibrosis, 28 patients had intermediate values, and 46 patients had advanced fibrosis. Serum gremlin 1 levels of the different subgroups did not differ (Figure 2b). The 36 patients with ultrasound-diagnosed liver cirrhosis had higher serum gremlin 1 levels than patients without liver cirrhosis (Figure 2c).

Multiple linear regression analysis showed that age, BMI, sex, diabetes, FIB-4 score, aminotransferases, GFR, creatinine, and platelet count did not predict serum gremlin 1 levels in patients with chronic HCV (F(10,83) = 1.698, *p* = 0.095).

### 3.3. The Relationship Between Serum Gremlin 1 Levels, Viral Load, and Genotype in Patients with HCV

**Figure 3:**
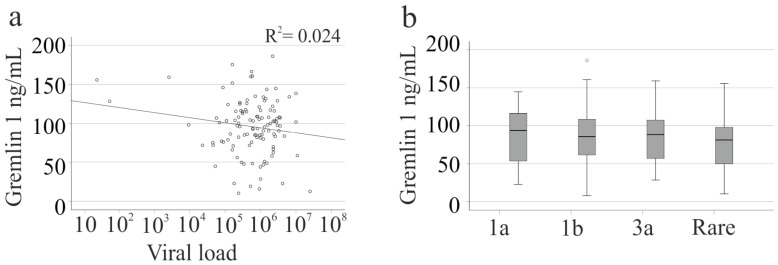
Serum gremlin 1 levels, viral load, and viral genotype: (**a**) Correlation of serum gremlin 1 with viral load. (**b**) Serum gremlin 1 levels in patients with chronic HCV stratified by viral genotype. A small circle above the box marks an outlier.

Serum gremlin 1 was not correlated with viral load (Figure 3a). The HCV genotypes included 1a (32 patients), 1b (56 patients), and 3a (16 patients), while rare genotypes were grouped together (10 patients). Serum gremlin 1 levels did not differ among the HCV genotypes (Figure 3b).

### 3.4. Association of Serum Gremlin 1 Levels with Inflammation, Lipoproteins, and Liver Function

In the patient cohort, serum gremlin 1 levels were not correlated with the model for end-stage liver disease (MELD) score, bilirubin, international normalized ratio (INR), and albumin. Serum gremlin 1 levels did not correlate with creatinine or the glomerular filtration rate (Table 2).

AST, ALT, and procalcitonin positively correlated with gremlin 1. Platelet and leukocyte counts, as well as C-reactive protein, were not associated with serum gremlin 1 levels. Low-density lipoprotein and high-density lipoprotein (HDL) did not correlate with gremlin 1 (Table 2).

### 3.5. Association of Serum Gremlin 1 Levels with Viral Cure

Direct-acting antiviral (DAA) therapy effectively eliminated HCV, resulting in a significant reduction in viral load [45]. Serum samples from 60 patients were available at the start of therapy, at 4 and 12 weeks during therapy, and at SVR12 (Table S1). All but two patients achieved optimal viral load suppression to <15 IU/mL [46]. Our PCR test method has a lower limit of detection (95% LoD) of 5.11 IU/mL in plasma or serum, and values below 12 IU/mL were designated as 1. The linear, quantifiable range begins at 12 IU/mL.

The characteristics of the patients who achieved SVR12 are summarized in Table 3. ALT, AST, and procalcitonin were lower, whereas albumin and LDL were higher at SVR12 than at pretreatment levels (Table 3). Viral load significantly decreased during DAA therapy (Table 3).

**Figure 4:**
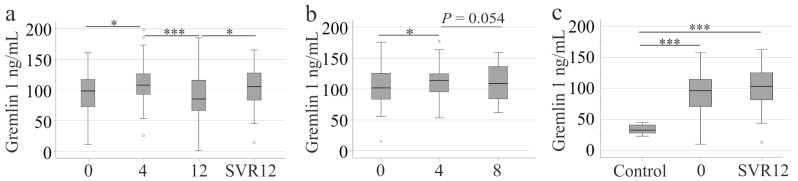
Serum gremlin levels during direct-acting antiviral therapy and comparison to controls at sustained virological response 12 (SVR12): (**a**) Gremlin 1 serum levels of 58 patients before therapy start (0), at 4 weeks after therapy start (4), at therapy end (12), and at SVR12. (**b**) Gremlin 1 serum levels of 22 patients, who are different from those 58 patients described before, where serum was available before therapy start (0), at 4 weeks after therapy start (4), and also at 8 weeks after therapy start. (**c**) Serum gremlin 1 levels of controls and 58 HCV patients before therapy (0) and at SVR12. Small circles above and below the boxes mark outliers. * *p* < 0.05, *** *p* < 0.001.

Serum gremlin 1 levels were increased at 4 weeks after therapy initiation compared with pre-treatment levels (Figure 4a).

Viral loads in the 58 patients at 4 weeks after therapy start did not correlate with gremlin 1 (r = 0.086, *p* = 0.521). Multiple linear regression analysis showed that age, BMI, sex, diabetes, FIB-4 score, aminotransferases, GFR, creatinine, and platelet count did not predict serum gremlin 1 levels in patients with chronic HCV at week 4 (F(10,89) = 0.371, *p* = 0.956).

Gremlin 1 significantly decreased from 4 to 12 weeks after therapy started. At the end of therapy (12 weeks) and at SVR12, gremlin 1 levels were similar to those observed in untreated HCV patients (Figure 4a).

To determine whether serum gremlin 1 levels returned to pre-treatment levels before 12 weeks of therapy, gremlin 1 concentrations were also measured in serum samples collected 8 weeks after treatment initiation. However, these samples were not collected systematically and were available only from patients who attended the clinic at that time, for reasons that were not documented. Serum samples from 22 patients, distinct from the 58 patients shown previously (Figure 4a), were collected before therapy and at 4 and 8 weeks after therapy initiation (serum samples at later time points were no longer available). In this subcohort, serum gremlin 1 was higher at 4 weeks than before treatment and declined until 8 weeks of therapy (Figure 4b).

Paired samples from 58 patients were available before therapy and at SVR12 (Figure 4a), and serum gremlin 1 did not differ between the cohorts and was higher in both cohorts than in healthy controls (Figure 4c).

At SVR12, serum from 85 patients could be analyzed, and serum gremlin 1 levels in patients with (31 patients) and without (54 patients) ultrasound-diagnosed liver cirrhosis were similar (*p* = 0.657).

## 4. Discussion

We demonstrate that serum gremlin 1 levels are elevated in patients with HCV compared to controls before treatment and at SVR12. However, these levels are not strongly associated with liver fibrosis or cirrhosis, either prior to therapy or at SVR12. Notably, serum gremlin 1 levels transiently increase during DAA therapy, suggesting a potential link to viral clearance, a hypothesis to be verified in future studies.

Gremlin 1 has been found to be upregulated in fibrotic rodent and human livers [23,28,29] and in the livers of patients with HCV [31]. Higher serum levels in HCV patients align with increased gremlin 1 protein in the livers of chronic HCV patients [31]. However, elevated gremlin 1 in liver fibrosis [23,28,29] does not translate into greatly higher serum levels, at least in patients with HCV. Serum gremlin 1 levels did not correlate with the MELD score or albumin; positive associations with aminotransferases were modest. When fibrosis was defined by SWE, HCV patients with F4 fibrosis before therapy had higher serum gremlin 1 levels than those with F0 fibrosis. Patients with ultrasound-diagnosed liver cirrhosis also had elevated serum gremlin 1 levels, indicating a link between liver cirrhosis and modestly elevated serum gremlin 1. At SVR12, serum gremlin 1 levels were similar in patients with and without cirrhosis; however, the smaller cohort size may have precluded detection of significant differences. These findings suggest that serum gremlin 1 is only weakly related to liver function and cannot be used to monitor the stages of liver fibrosis in patients with chronic HCV. However, noninvasive diagnosis of liver fibrosis and cirrhosis, as was used in our analysis, bears a risk of misclassification [47], and analysis in patients with biopsy-confirmed liver cirrhosis is required.

HCV infection can contribute to the development of diabetes and liver steatosis [48], and gremlin 1 has been shown to induce fatty liver [25]. In our cohort, patients with and without steatosis had similar serum gremlin 1 levels. Metabolic dysfunction-associated steatotic liver disease (MASLD) is prevalent in the general population, particularly among HCV patients [49]. However, diagnosing MASLD in patients with HCV infection is challenging [49], and our study did not distinguish virus-induced fatty liver disease from metabolic liver disease. Consequently, we can only conclude that, regardless of the cause, liver steatosis is not associated with altered serum gremlin 1 levels.

Moreover, associations between serum gremlin 1 levels and measures of insulin resistance were observed [22]. In our cohort, however, diabetes was not associated with higher serum gremlin 1 levels.

Other studies have described gremlin’s role in inflammation and shown that platelets release gremlin 1 upon activation [50]. However, serum gremlin 1 did not correlate with platelet number, leukocyte count, or C-reactive protein, and the correlation with procalcitonin was weak. Some of our patients had low platelet counts. The cause of thrombocytopenia, especially if associated with portal hypertension or viral infection itself, was not further analyzed in this cohort. Overall, serum gremlin 1 was not associated with thrombocytopenia.

HCV is classified into eight genotypes [51], and genotype 1 is the most prevalent in Europe, with subtypes 1a and 1b being particularly common [52]. In our study, 88 patients were infected with either 1a or 1b. It is important to note that HCV genotypes exhibit differences in clinical characteristics. For example, insulin resistance and liver steatosis are more frequently observed in infections caused by genotypes 1 and 3 [53]. However, serum gremlin 1 levels were comparable among patients infected with genotypes 1a, 1b, or 3a. Serum gremlin 1 levels were not associated with viral load, and HCV elimination by DAA therapy did not alter gremlin 1 levels in the blood.

Changes associated with HCV that persist after viral cure have been described. Elevated inflammatory cytokine levels in chronic HCV declined during DAA treatment but did not reach levels comparable to those in healthy controls [10]. Cytokines such as IL-4 and interferon gamma, which were repressed in HCV, did not normalize at SVR12 [10]. The profibrotic protein Diaphanous-Related Formin 1 was higher in extracellular vesicles from patients with HCV than in controls, and it did not change in patients after six months of DAA therapy, although all patients achieved SVR [54]. Non-invasive fibrosis scores such as SWE and FIB-4 show a short-term decrease after treatment, which may reflect improvements in inflammation rather than fibrosis [55]. Liver fibrosis scores, as defined by biopsies taken before treatment and approximately three years after achieving SVR, improved rather than worsened [56]. Ongoing regression of fibrosis, as documented by non-invasive tests, is observed at 48, 96, and 144 weeks after HCV cure across all fibrosis stages. Whether elevated gremlin 1 levels in patients with HCV after viral clearance contribute to medium-term persistence of fibrosis [55] requires further study.

A multicenter retrospective cohort study in South Korea, including over 10,000 patients, showed that the DAA group had a lower risk of HCC, hepatic decompensation, and mortality than the untreated group [57]. However, HCV patients who achieved an SVR after DAA treatment have a higher incidence of adverse clinical outcomes (5-year cumulative rates of liver cirrhosis, decompensated cirrhosis, HCC, and all-cause mortality) compared to never-infected controls [58]. A European study including 2335 patients identified compensated advanced chronic liver disease, defined by liver stiffness measurement of >10 kPa, as a risk for the development of HCC even after more than 3 years of viral cure [59]. Fibroblast-released gremlin was found to promote HCC development [33], and gremlin 1 was shown to induce cell proliferation and migration [60]. Persistent gremlin 1 elevation after HCV cure could be associated with a higher risk for HCC. However, the role of elevated serum gremlin 1 in this context is unclear. Moreover, studies analyzing serum gremlin 1 levels over longer follow-up periods are needed.

Notably, gremlin 1 levels transiently increased 4 weeks after therapy started. Levels before therapy and at 8 and 12 weeks after therapy started did not differ from pretreatment levels, nor did levels at SVR12. All ELISA assays performed were ordered together, eliminating inter-batch variation. Serum samples collected at different time points were analyzed on the same assay plates to minimize technical variability. Platelet number, which can contribute to serum gremlin 1 levels [50], was similar at all time points. Notably, there was a non-significant decrease in platelets at 4 weeks after the start of therapy. Regression to the mean resulting from an unusually high measurement at week 4, or from low levels at baseline, week 12, or SVR12, is unlikely to explain this observation. Higher gremlin 1 levels at 4 weeks after therapy started were observed in two independent cohorts and remained evident when gremlin-1 was analyzed across all available serum samples (114 patients before therapy, 126 at week 4, 106 at week 12, and 85 at SVR12; Figure S2). The routine laboratory markers analyzed in our patient cohort did not show a transient increase at week 4 after the start of DAA therapy, and the relationship between gremlin 1 transient elevation and DAA therapy remains unresolved.

In patients with HCV, transient increases in the expression of dual-specificity phosphatase 1, JunB, and Fos in T cells have been reported at week 4 of DAA treatment. These levels were comparable to those in normal control subjects, indicating an early normalization of T cell function that was not maintained following treatment [61]. Sato et al. reported an early, temporary increase in serum uric acid levels during sofosbuvir and ribavirin therapy [62]. Currently, the function of transiently elevated gremlin 1 levels during DAA therapy remains unexplained, nor is it clear which pathways mediate this increase.

Gremlin 1 is expressed in adipose tissue, and higher serum levels in obese individuals have been suggested to correlate with increased fat mass [22,63]. However, in patients with HCV, serum gremlin 1 did not correlate with body mass index. Furthermore, serum gremlin 1 did not correlate with age or differ between sexes.

This study cannot provide evidence that serum gremlin 1 has diagnostic value in patients with HCV. There are only minor associations with liver fibrosis or measures of liver disease, and serum gremlin 1 levels do not change upon viral cure. Whether the persistence of high serum gremlin 1 levels after HCV eradication is related to more adverse outcomes or the development of HCC is a matter of future studies.

This study has several limitations. First, liver biopsy specimens were unavailable; therefore, it was not possible to determine whether hepatic gremlin 1 is induced in chronic HCV infection and/or advanced fibrosis. Second, fibrosis staging and the diagnosis of liver cirrhosis were based on non-invasive methods, which may have resulted in some degree of misclassification. Third, the longitudinal cohort sample size was relatively small. In addition, serum samples were collected only shortly after viral eradication, and no long-term follow-up was performed. Fourth, the controls were purely described, and only age and sex were known. This limits the validity of comparisons between controls and patients’ gremlin 1 levels. Fifth, this observational study cannot provide functional insights into the regulation of gremlin 1 in chronic HCV or during DAA therapy. Finally, this was a single-center study, which may limit the generalizability of the findings.

## 5. Conclusions

In summary, this analysis indicates that serum gremlin 1 is not a reliable marker of liver fibrosis stage in patients with chronic HCV infection, either before or after viral eradication. However, its persistent elevation following SVR12 suggests that it may provide clinically relevant information beyond fibrosis assessment. The sustained increase in serum gremlin 1 at SVR12 despite successful DAA therapy warrants further investigation to determine whether this biomarker normalizes over longer follow-up or reflects residual disease activity with an increased risk of adverse long-term outcomes after HCV cure.

## Figures and Tables

**Table 1:** Gender distribution, age, body mass index, model for end-stage liver disease score, and laboratory parameters of the 114 patients before therapy started. If laboratory values were not obtained for all patients, the number (n) of patients for whom these measures were known is reported in uppercase.

Parameter	Before Therapy
Gender (F/M)	52/62
Body mass index kg/m^2^	26.6 (18.4–40.4) ^95^
Age years	57 (24–82)
MELD Score	7 (6–19)
ALT U/L	71 (22–287)
AST U/L	55 (14–1230)
Bilirubin mg/dL	0.6 (0.2–4.3)
Albumin g/L	37.5 (19.0–45.5)
INR	1.1 (0.9–2.3)
Creatinine mg/dL	0.8 (0.1–1.5)
GFR mL/min	98 (37–161)
Leukocyte number/L	6.3 (2.2–12.3)
CRP mg/L	2.9 (2.9–29.9)
PCT ng/mL	0.07 (0.0–11.02)
Platelet number/nL	170 (38–364)
HDL mg/dL	52 (19–103) ^107^
LDL mg/dL	93 (31–204) ^107^
Viral load IU/mL	7 × 10^5^ (23–25 × 10^6^)

ALT, alanine aminotransferase; AST, aspartate aminotransferase; CRP, C-reactive protein; GFR, glomerular filtration rate; HDL, high-density lipoprotein; INR, international normalized ratio; LDL, low-density lipoprotein; MELD, model for end-stage liver disease; PCT, procalcitonin.

**Table 2:** Spearman correlation coefficients and *p*-values for the correlation of gremlin 1 with routine laboratory measures.

Parameter	Correlation Coefficient	*p*-Value
MELD Score	−0.120	0.202
ALT U/L	0.185	0.048
AST U/L	0.202	0.031
Bilirubin mg/dL	0.074	0.432
Albumin g/L	−0.012	0.897
INR	−0.128	0.174
Creatinine mg/dL	0.067	0.482
GFR mL/min	0.013	0.890
Leukocyte number/L	0.109	0.246
CRP mg/L	0.089	0.344
PCT ng/mL	0.211	0.025
Platelet number/nL	−0.141	0.135
HDL mg/dL	0.025	0.800
LDL mg/dL	−0.154	0.113

**Table 3:** Gender distribution, age, body mass index, model for end-stage liver disease score, and laboratory parameters of the 58 patients with serum available before and during therapy with DAAs, as well as at SVR12. If laboratory values were not obtained for all patients, the number (n) of patients for whom these measures were known is reported.

Parameter	Before Therapy	4 Weeks	8 Weeks	SVR12	*p*-Value
Gender (F/M)	28/30	28/30	28/30	28/30	>0.05
BMI kg/m^2^	25.7 (18.4–40.4) ^48^	25.7 (18.4–40.4) ^48^	25.7 (18.4–40.4) ^48^	25.7 (18.4–40.4) ^48^	>0.05
Age years	57 (27–79)	57 (27–79)	57 (27–79)	57 (27–79)	>0.05
MELD Score	7 (6–13)	7 (6–13)	7 (6–13)	7 (6–13)	>0.05
ALT U/L	72 (22–240)	26 (7–85)	26 (8–88)	23 (6–135)	<0.001
AST U/L	53 (14–208)	24 (10–96)	22 (7–161)	21 (10–76)	<0.001
Bilirubin mg/dL	0.6 (0.3–2.0)	0.7 (0.1–2.8)	0.6 (0.1–2.8)	0.6 (0.4–2.8)	>0.05
Albumin g/L	36.8 (28.3–45.5)	37.8 (28.9–45.5)	38.3 (29.2–44.4)	39.2 (26.1–47.7)	<0.001
INR	1.1 (0.9–1.5)	1.1 (0.9–1.5)	1.1 (0.9–1.4)	1.1 (0.1–1.5)	>0.05
Creatinine mg/dL	0.8 (0.1–1.2)	0.8 (0.2–1.3)	0.7 (0.1–1.9)	0.8 (0.1–1.3)	>0.05
GFR mL/min	97 (47–161)	97 (42–140)	95 (33–126)	96 (41–125)	>0.05
Leukocyte number/L	6.0 (2.2–11.5)	6.4 (2.0–12.8)	6.3 (2.6–12.9)	6.2 (1.9–12.8)	>0.05
CRP mg/L	2.9 (2.9–29.9)	2.9 (2.9–19.8)	2.9 (2.9–19.1)	2.9 (2.9–20.4)	>0.05
PCT ng/mL	0.07 (0.01–11.02)	0.05 (0.01–11.89)	0.03 (0.01–0.16)	0.03 (0.00–0.14)	<0.001
Platelet number/nL	165 (38–364)	148 (31–384)	177 (37–379)	169 (38–391)	>0.05
HDL mg/dL	53 (22–103) ^53^	56 (22–102) ^56^	56 (23–96) ^54^	52 (23–85) ^57^	>0.05
LDL mg/dL	85 (31–204) ^53^	118 (44–242) ^56^	112 (46–251) ^54^	116 (47–243) ^53^	<0.001
Viral load IU/mL	7 × 10^5^ (23–25 × 10^6^)	12 (1–1700)	1 (1–26)	1 (1–10)	<0.001

ALT, alanine aminotransferase; AST, aspartate aminotransferase; BMI, body mass index; CRP, C-reactive protein; GFR, glomerular filtration rate; HDL, high-density lipoprotein; INR, international normalized ratio; LDL, low-density lipoprotein; MELD, model for end-stage liver disease; PCT, procalcitonin.

## Data Availability

Data supporting reported results are included in the manuscript.

## Supplementary Materials

The following supporting information can be downloaded at: https://www.mdpi.com/article/10.3390/v18070773/s1. Figure S1: Description of the study cohort. Figure S2: Serum gremlin 1 in all available serum samples. Table S1: Gender distribution, age, body mass index, model for end-stage liver disease score, and laboratory parameters of the 60 patients with serum available before and during therapy with DAAs, as well as at SVR12.

## Abbreviations

The following abbreviations are used in this manuscript:

ALT	Alanine aminotransferase
AST	Aspartate aminotransferase
BMI	Body mass index
CRP	C-reactive protein
DAA	Direct-acting antivirals
FIB-4	Fibrosis-4
GFR	Glomerular filtration rate
HCC	Hepatocellular carcinoma
HCV	Hepatitis C Virus
HDL	High-density lipoprotein
INR	International normalized ratio
LDL	Low-density lipoprotein
MASLD	Metabolic dysfunction-associated steatotic liver disease
MELD	Model for end-stage liver disease
PCT	Procalcitonin
SVR12	Sustained virological response 12
SWE	Shear wave elastography
