# Supplementary material for: Serum Gremlin 1 Remains Elevated Following Successful Direct-Acting Antiviral Therapy for Chronic Hepatitis C
Source: Viruses. 2026 Jul 14;18(7):773. doi: 10.3390/v18070773 (PMC13431586; doi:10.3390/v18070773)
Supplement: Supplementary file 1 [file viruses-18-00773-s001.zip › viruses-4383648-supplementary.pdf]

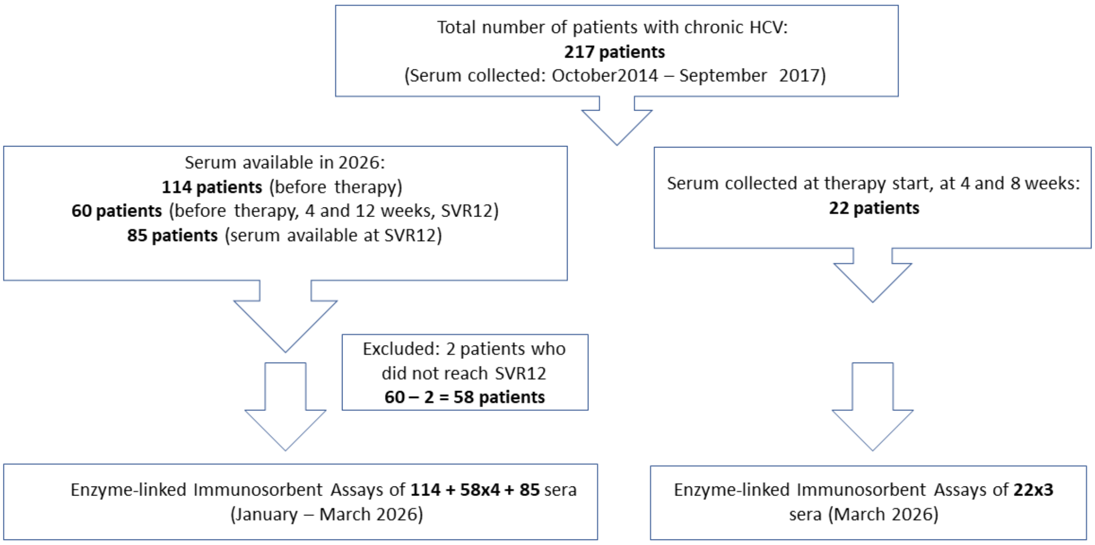

Figure S1: Description of the study cohort. Because this cohort has been used in previous studies (which cannot be cited due to the self-citation limit), the sample size available at different time points varies.

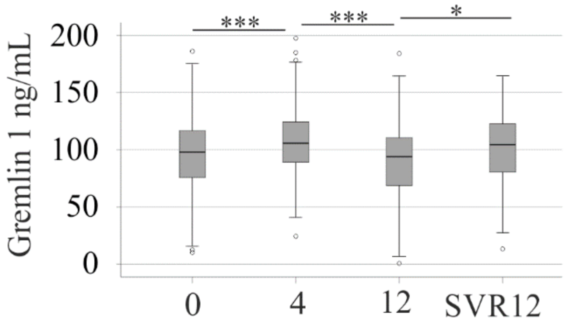

Figure S2: Serum gremlin 1 in all available serum samples (114 patients before therapy, 126 at week 4, 106 at week 12, and 85 at SVR12.

Table S1. Gender distribution, age, body mass index, model for end-stage liver disease score, and laboratory parameters of the 60 patients with serum available before and during therapy with DAAs, as well as at SVR12. If laboratory values were not obtained for all patients, the number (n) of patients for whom these measures were known is reported.

**Parameter**	**Before therapy**	**SVR12 (all patients)**	***P*-value**
Gender (F / M)	29 / 31	29 / 31	> 0.05
BMI kg/m^2	25.7 (18.4 - 40.4)[50]	25.7 (18.4 - 40.4)[50]	> 0.05
Age years	57 (27 - 79)	57 (27 - 79)	> 0.05
MELD Score	7 (6 - 13)	7 (6 - 15)	> 0.05
ALT U/L	72 (22 - 240)	25 (6 - 135)	< 0.001
AST U/L	56 (14 - 1230)	22 (10 - 1390)	< 0.001
Bilirubin mg/dL	0.6 (0.2 - 2.0)	0.6 (0.4 - 2.8)	> 0.05
Albumin g/L	36.7 (28.3 – 45.5)	39.2 (26.1 – 47.7)	< 0.001
INR	1.1 (0.9 – 1.5)	1.1 (0.1-1.5)	> 0.05
Creatinine mg/dL	0.8 (0.1 - 1.2)	0.8 (0.1 - 1.3)	> 0.05
GFR mL/min	97 (47 - 161)	96 (41 - 127)	> 0.05
Leukocyte number/L	6.0 (2.2 - 12.3)	6.2 (1.9 - 12.8)	> 0.05
CRP mg/L	2.9 (2.9 - 29.9)	2.9 (2.9 - 20.4)	> 0.05
PCT ng/ml	0.07 (0.0 - 11.02)	0.03 (0.01 - 0.14)	0.002
Platelet number/nL	165 (38 - 364)	169 (38 - 391)	> 0.05
HDL mg/dL	52 (22 - 103) [55]	51 (23 - 85) [57]	> 0.05
LDL mg/dL	85 (31 - 204) [55]	117 (47 - 243) [56]	0.01
Viral load IU/mL	$7x10^5$ (23 -25 x 10^6)	1 (1 - 35 x 10^5)	< 0.001
